# Diagnostic and Therapeutic Biomarkers in Glioblastoma: Current Status and Future Perspectives

**DOI:** 10.1155/2017/8013575

**Published:** 2017-02-20

**Authors:** Wojciech Szopa, Thomas A. Burley, Gabriela Kramer-Marek, Wojciech Kaspera

**Affiliations:** ^1^Department of Neurosurgery, Medical University of Silesia, Regional Hospital, Sosnowiec, Poland; ^2^Division of Radiotherapy and Imaging, The Institute of Cancer Research, London, UK

## Abstract

Glioblastoma (GBM) is a primary neuroepithelial tumor of the central nervous system, characterized by an extremely aggressive clinical phenotype. Patients with GBM have a poor prognosis and only 3–5% of them survive for more than 5 years. The current GBM treatment standards include maximal resection followed by radiotherapy with concomitant and adjuvant therapies. Despite these aggressive therapeutic regimens, the majority of patients suffer recurrence due to molecular heterogeneity of GBM. Consequently, a number of potential diagnostic, prognostic, and predictive biomarkers have been investigated. Some of them, such as IDH mutations, 1p19q deletion, MGMT promoter methylation, and EGFRvIII amplification are frequently tested in routine clinical practice. With the development of sequencing technology, detailed characterization of GBM molecular signatures has facilitated a more personalized therapeutic approach and contributed to the development of a new generation of anti-GBM therapies such as molecular inhibitors targeting growth factor receptors, vaccines, antibody-based drug conjugates, and more recently inhibitors blocking the immune checkpoints. In this article, we review the exciting progress towards elucidating the potential of current and novel GBM biomarkers and discuss their implications for clinical practice.

## 1. Introduction

**Figure 1:**
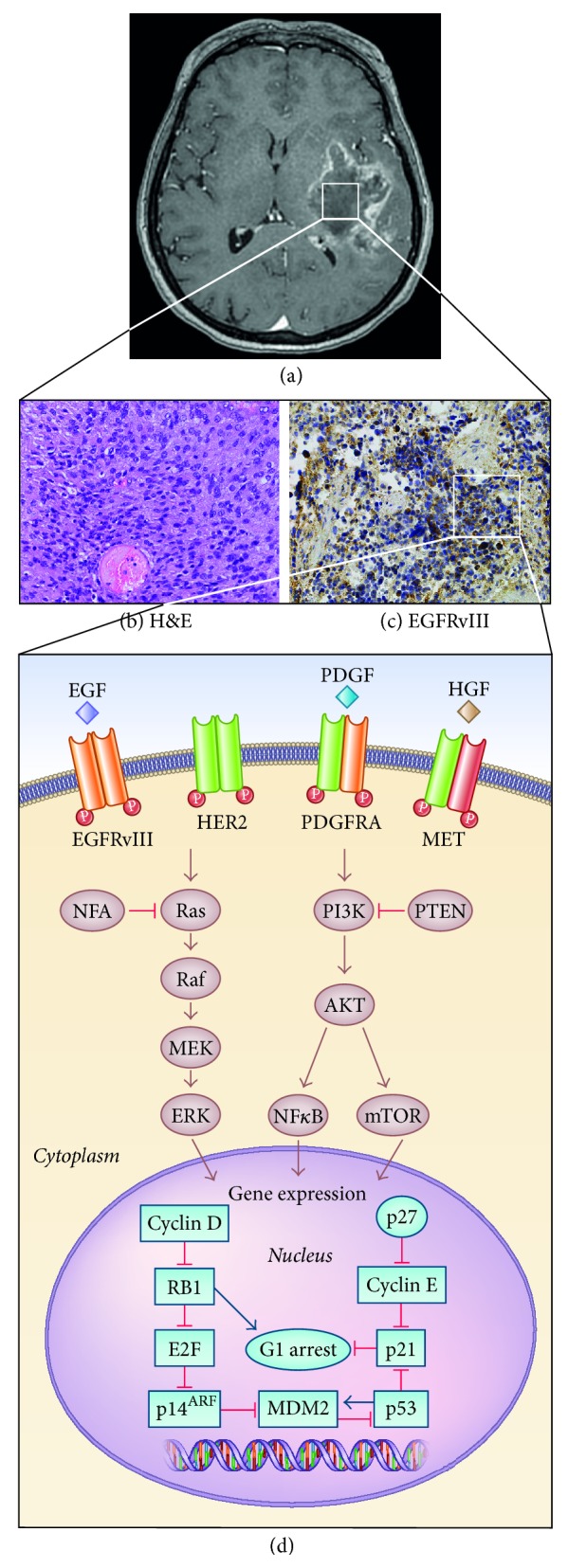
Glioblastoma characterization. (a) Axial contrast-enhanced T1-weighted MR image shows a large enhancing mass in the left temporal lobe in a 54-year-old woman diagnosed with GBM. (b, c) GBM formalin-fixed paraffin-embedded sections stained for H&E and EGFRvIII. (d) Frequent alterations in critical signaling pathways found in GBM.

Glioblastoma (GBM) is a primary neuroepithelial tumor of the central nervous system, characterized by an extremely aggressive clinical phenotype that has arisen from inter- and intrapatient genomic and histopathological diversity (Figure 1; Table 1). GBM is the most common of malignant primary brain tumors in adults. It accounts for 12% to 15% of all intracranial tumors and about 50% of astrocytic tumors.

Patients with GBM have a poor prognosis of just 12–15 months following standard therapy, with only 3–5% of patients surviving up to 5 years after diagnosis [1, 2]. The most favorable prognostic factors include younger age at diagnosis (<50 years), a Karnofsky Performance Status of at least 70 points, and the tumor being located in a noneloquent area of the brain [3]. The current GBM treatment standards include maximal resection (complete resection is achieved extremely rarely due to the diffusely infiltrative nature of these tumors) followed by radiotherapy with concomitant and adjuvant therapies, for example, temozolamide (TMZ). If there is progression, bevacizumab against circulating vascular endothelial growth factor (VEGF) is widely used, more recently also in combination with lomustine (CCNU) [4]. Despite these aggressive therapeutic regimens the majority of patients suffer recurrence due to the molecular heterogeneity of GBM tumors and penetration of therapeutic agents through the blood-brain barrier (BBB). Both of these factors affect treatment response and prognosis leading to acquired tumor resistance in GBM patients. However, recent developments in next-generation sequencing methods have led to identification of specific molecular signatures of GBM that allow for better understanding of the molecular pathogenesis of this disease [5]. Consequently, a number of potential diagnostic, prognostic, and predictive biomarkers have been proposed. Diagnostic biomarkers enable more accurate tumor classification; prognostic biomarkers inform about a likely cancer outcome (e.g., disease recurrence, disease progression, and overall survival) and predictive biomarkers facilitate patient management by helping to tailor the treatment strategy to patient-specific biology. There are some molecular markers still under evaluation, but several are commonly tested as part of the routine clinical interrogation of GBM patients including O^6^-methylguanine DNA methyltransferase (MGMT), isocitrate dehydrogenase (IDH), epidermal growth factor receptor (EGFR), VEGF, tumor suppressor protein TP53, phosphatase and tensin homolog (PTEN), p16INK4a gene, phospholipid metabolites, cancer stem cells, and recently also imaging biomarkers (Table 2). Importantly, detailed characterization of these molecular signatures has facilitated a more personalized therapeutic approach and contributed to the development of a new generation of anti-GBM therapies such as small molecular inhibitors targeting growth factor receptors, vaccines, antibody-based drug conjugates, and more recently inhibitors blocking the immune checkpoints [6].

The aim of this article is to review the exciting progress towards elucidating the potential of diagnostic, prognostic, and predictive biomarkers of GBM and discuss their implications for clinical practice.

## 2. Glioblastoma Multiforme: A New Look

Gliomas have historically been classified and treated according to the World Health Organization (WHO) criteria, which are determined by histopathological examination, for example, nuclear atypia, cellular pleomorphism, mitotic activity, vascular thrombosis, microvascular proliferation, and necrosis [7]. In the official reclassification of Tumor Types of the Central Nervous System, published by WHO on May 9, 2016, the GBMs are listed in the group of diffuse astrocytic and oligodendroglial tumors which reflect their highly malignant behavior [8].

Clinically, most patients present de novo grade IV lesions (primary GBMs), whereas only a small fraction of patients (5–10%) show progression from less aggressive WHO grade II diffuse astrocytomas and WHO grade III anaplastic astrocytomas (secondary GBMs) [9].

Medical onset and progression of primary GBMs vary from those seen in secondary GBMs, with the latter being typically diagnosed at a younger age (45 versus 62 years), having longer clinical history (16.8 versus 6.3 months) and, although they are histologically largely indistinguishable, having a better prognosis in terms of survival (7.8 versus 4.7 months) [10].

Importantly, these two clinical presentations have distinct molecular signatures. For example, primary GBMs frequently present amplification/mutations of the EGFR gene (36–60% of primary and 8% of secondary tumors), PTEN mutation (25% of primary versus 4% secondary tumors), and CDKN2A-p16^INK4a^ deletion (31–78% of primary versus 8% secondary tumors) [10]. Additionally, there are genetic aberrations that are expressed more frequently in secondary GBMs including TP53 mutations (28% of primary versus 65% of secondary tumors) [10], MGMT promoter methylation (36% of primary versus 75% of secondary tumors) [11], and IDH1 mutations (5% of primary versus 75% of secondary tumors) [12]. While histopathological analysis of gliomas has formed the basis of diagnosis and treatment up to this point, the widespread implementation of sequencing and profiling technologies has resulted in more comprehensive analysis of the molecular aberrations underlying gliomagenesis, as well as providing insights into their biological heterogeneity.

The more recent analysis of GBMs by The Cancer Genome Atlas Research Network (TCGA) highlighted the most frequent alterations in GBM genes, including amplification of EGFR and platelet-derived growth factor receptor alpha (PDGFRA); mutation of TP53, phosphatidylinositol-4,5-bisphosphate 3-kinase A (PIK3CA), PTEN, IDH1, RB1, and TERT promoter; and deletions of PTEN, CDKN2A/B, and MGMT, as well as alterations in chromatin remodeling genes. Based on these dominant gene expression patterns, four transcriptional subclasses of GBMs have been identified: classical, proneural, mesenchymal, and neural [13, 14]. Each of these subtypes is associated with specific genetic and epigenetic alterations. The classical subtype is characterized by the loss of chromosome 10 and amplification of chromosome 7 with coexisting EGFR amplification/mutation, impaired level of proapoptotic proteins, mitogen-activated protein kinase (MAPK), and Notch1 and Notch3 proteins [14, 15]. The proneural variant is associated with PDGFRA, CDK6, CDK4 MET, and frequent IDH1 mutations, activation of the phosphatidylinositol 3-kinase (PI3K), and inhibition of the translation repressor 4EBP1 [14].

Mesenchymal GBMs frequently show deletions and silencing mutations of NF1 on chromosome 17q11.2 and point mutations in PTEN, activation of MAPK pathway, and downregulation of mammalian target of rapamycin (mTOR) signaling.

Less is known about the neural tumors, which are characterized by the expression of neurofilament light polypeptide, synaptotagmin I, and overexpression of EGFR [16]. In terms of prognosis, no difference was found between the classical, mesenchymal, and neural subtypes. However, the proneural subtype was associated with onset at a younger age and prolonged survival time [14]. This has been attributed to mutations in the IDH1 gene, which are exclusively linked with the proneural phenotype and grade II/III of astrocytic and oligodendroglial tumors (72–100%) along with secondary glioblastomas (85%). While primary GBMs could be of any subtype and mutations in IDH1 are rarely found in these tumors (5%) [17].

Classification and subclassification of GBMs is not always easy. Recent studies analyzing expression signatures of single cells within GBM samples showed substantial intratumoral heterogeneity of expression subclasses within each tumor. Sattoriva et al. examined genome-wide somatic copy number levels in 38 fragments derived from 11 patients with GBM. Although the fragments from the same patient shared a common gene profile, they displayed a significant variety of copy number alterations that were present in only a subset of fragments. Moreover, using gene expression arrays, they found that in 6 out of 10 cases the fragments from the same tumor mass were classified into at least 2 different GBM subgroups, which indicated that tumor clones with different phenotypic profiles are present within the same malignancy [18]. This may explain the difficulties associated with oncologic biomarker validation and contribute to an incorrect selection of patients for targeted therapies, treatment failure, or drug resistance [19].

Additionally, GBM exhibit heterogeneity at the cellular level, with a small subpopulation of tumor cells harboring stem-like properties. These GBM stem cells (GBSC) are capable of self-renewal and differentiation into neuronal, macroglial, and mixed neuronal/astroglial phenotypes [20]. Recent genome-wide transcriptional analysis identified two phenotypically different subtypes of GBSC, namely, proneural and mesenchymal, which also correlate well with corresponding proneural and mesenchymal signatures in GBMs [21, 22]. Mesenchymal GBSC (35–40% of cases) similarly to mesenchymal GBMs, display a more aggressive phenotype and are more resistant to radiation as compared to proneural GBSC. Moreover, GBSC possess unique surface markers (e.g., CD133, CD15, and ALD1A3) and modulate characteristic signaling pathways to promote tumorigenesis (e.g., hedgehog and Notch) [23]. Interestingly, these GBSC have the ability to shift phenotypic features from one subtype to another when put under increased cellular stress (e.g., radiation treatment) [22–24] and transdifferentiate into tumor microenvironment cells such as endothelial cells and pericytes, providing more favorable conditions for GBM growth [25].

## 3. A Highway to Hell: Molecular Pathways and Genetic Aberrations Found in GBM

Tumor growth in GBM cells is facilitated by high expression of cell surface membrane receptors that control the intracellular signal transduction pathways regulating proliferation and cell cycle abnormalities including an increase in DNA repair proteins and abnormal cell death pathways [26, 27]. An integrated analysis of the genetic alterations, performed by the TCGA research network, confirmed that the most commonly disrupted signaling cascades in GBM include changes in pathways related to receptor tyrosine kinase (RTK) signaling through the RAS/MAPK (mitogen-activated protein kinase) and PI3K/AKT/mTOR, along with the cell cycle-regulating retinoblastoma (RB) tumor suppressor and p53 pathways.

## 4. RTK Signaling in GBM

Mutations or amplifications of RTK including EGFR, PDGFRA, basic fibroblast growth factor receptor 1 (FGFR-1), and insulin-like growth factor receptor (IGFR-1) are present in more than 80% of primary GBM [28]. These structurally related proteins coordinate a complex signaling network that drives and regulates many cellular processes. In gliomas the two main signaling pathways utilized by RTK are the RAS/RAF/MAPK pathway that leads to cellular proliferation, differentiation, and migration and the PI3K/AKT/mTOR pathway that primarily serves to promote cell proliferation and survival through progression of the cell cycle and inhibition of apoptosis [28]. The activity of PI3K is regulated by the tumor suppressor gene PTEN that is a negative regulator of this pathway [29]. Loss of PTEN, found in approximately 36% of gliomas, may result in dramatic upregulation of this pathway and be a major source of resistance to EGFR therapies [30].

EGFR mutations, rearrangements, alternative splicing, and focal amplifications are the most frequent genetic alterations, occurring in nearly 57% of GBM tumors [31]. EGFR amplification can be found, nearly exclusively, in patients with a classical subtype of GBM and is very rare in secondary GBMs [16]. However, the role of EGFR amplification as a prognostic biomarker has yielded conflicting results. There are reports showing no association with overall survival (OS) in patients, others showing a negative impact, and some even indicating a favorable impact on patient survival [32–36]. Unfortunately, despite the high frequency of EGFR gene amplification, EGFR inhibitors (e.g., gefitinib and erlotinib) have not been successfully brought into clinical trials for patients with GBMs [37–39].

The lack of a meaningful response may be due to the relatively poor penetration of these drugs through BBB, acquired resistance promoting mutations in the targeted RTKs, and intratumoral heterogeneity in GBM tumors [40, 41]. In addition, around 50% of patients with EGFR amplification harbor EGFRvIII mutation, which results from an in-frame deletion of exons 2–7 and leads to constitutive and ligand independent receptor activity [42]. However, EGFRvIII prognostic relevance is still controversial, for instance, Shinojima et al. have shown that EGFRvIII expression in the presence of EGFR amplification is a strong indicator of poor survival and prognosis [43]. On the other hand, Montano et al. prospectively analyzed the relationship between EGFRvIII expression and OS in patients with newly diagnosed GBM treated with gross total resection and standard radiochemotherapy (TMZ). Notably in this case, EGFRvIII identified that patients had significantly longer OS. Furthermore, association of EGFRvIII/Ki67 of 20% or less, EGFRvIII/normal PTEN, and EGFRvIII/methylated MGMT allowed identification of subgroups in GBM patients with better prognosis [44].

Although EGFRvIII seems to be a well-defined drug target the clinical trials with tumor vaccine rindopepimut have demonstrated immunologic effect and apparent clinical benefit only in early phase trials. Tests of this vaccine in randomized, placebo-controlled phase III studies failed to show survival benefit [45]. Nevertheless, EGFR still remains an attractive molecular target and current clinical trials are focusing on testing new inhibitors that are more potent and specific to the GBM mutations and introducing mechanism-based combination therapies [40, 46, 47].

When it comes to other RTK alterations, in a smaller proportion of secondary GBMs (13%), high-level amplification of the PDGFRA has been detected, and nearly half of these tumors also contained amplification and/or mutation of EGFR [31, 48]. Despite PDGFRA being strongly associated with GBM, an anti-PDGFRA therapy using glivec resulted in only a limited clinical response [49].

Although much less frequent in GBMs, alterations such as mesenchymal-epithelial transition factor (c-Met) amplification and FGFR mutations have been reported in 2% of the analyzed GBMs [50, 51].

All these activating genetic aberrations can occur simultaneously in multiple RTKs within individual GBM and concurrently express mutations in downstream components of growth factor receptor pathways. The PI3K/AKT/mTOR, the most powerful oncogenic pathway in GBM, can be activated by mutations in either the catalytic (PIK3CA) or regulatory (PIK3R1) domains of PI3K [52]. The TCGA study found that almost 10% of the GBMs had mutations in the PIK3R1, which has not been found to be frequently expressed in any other cancer [53]. Furthermore, it has been reported that AKT classification can be a predictive marker that identifies a subset of GBM patients responding to carmustine (BCNU)/CCNU and PI3K/AKT/mTOR pathway inhibitors [54]. More recent studies have revealed that the tumor suppressor gene NF1, that encodes neurofibromin (RAS negative regulator), is mutated/deleted in 15% to 18% of primary GBMs (mesenchymal subclass) [53, 55].

## 5. TP53/MDM2/p14^ARF^ Pathway

The TP53 tumor suppressor gene, at chromosome 17q13.1, encodes a p53 protein that regulates target genes involved in (i) cell cycle arrest in the G1 and/or G2 phase of cell cycle, (ii) cell death and differentiation, (iii) DNA repair, and (iv) neovascularization [56–58]. TP53 may be inactivated indirectly, as a result of mutation or deletion, or directly due to damage of cooperating genes [59]. MDM2 oncoprotein negatively regulates p53 activity through the ubiquitination and proteasomal degradation of p53. In turn, the p14^ARF^ protein functionally antagonizes MDM2 and, thus, prevents the silencing of p53 [60]. Initially, TP53 mutations have been associated with secondary GBMs rather than with primary (65% versus 28%) [10], but recent TCGA data has reported TP53 mutations in many primary GBMs. The overall frequency of genetic alterations in the TP53/MDM2/p14^ARF^ pathway was found in 87% of GBMs, in 35% through TP53 mutations or homozygous deletion, in 14% due to MDM2 amplification, and in 49% as a result of p14^ARF^ homozygous deletion or mutation [53].

## 6. p16^INK4a^/CDK4/RB1 Pathway

The RB1 protein controls progression through G1 into the S-phase of the cell cycle. The CDKN2A (p16^INK4a^) protein binds to CDK4 cyclin and inhibits the CDK4/cyclin D1 complex that prevents cell cycle transition from G1 to S-phase [61]. Therefore, loss of normal RB1 function may result from altered expression of any of the p16INK4a, CDK4, or RB1 genes.

Inactivation of this pathway is commonly observed in both primary and secondary GBMs. While mutations in RB1 are not common (11%), genes encoding its upstream regulators are frequently altered, in particular mutations and deletions of CDKN2A/p16 (52%) and amplification of CDK4 (18%) [62]. Despite frequent occurrence of these defects none of them have been identified as a useful prognostic biomarker in GBMs [63].

## 7. IDH Mutations

The IDH1 and IDH2 genes encode two critical metabolic enzymes: isocitrate dehydrogenase 1 (present in the peroxisomes and cytosol) and isocitrate dehydrogenase 2 (present in the mitochondria). These proteins catalyze the oxidative carboxylation of isocitrate to alpha-ketoglutarate, which results in the production of NADPH in the citric acid (Krebs) cycle [64, 65]. Mutations of these IDH genes promote reactions that generate the oncometabolite 2-hydroxyglutarate (2-HG) [66, 67]. In gliomas the most frequent missense mutations in IDH genes are present at the 132 residue in IDH1 (85%) and at 172 in IDH2 (3%) [12, 68]. They have been mainly found in secondary GBMs (73%–85%), along with grades II and III astrocytic and oligodendroglial tumors (72–100%) but appear to be rare or absent in primary GBMs (5%) [12, 69]. Several studies have reported that the presence of IDH mutations in diffuse glioma is associated with younger age (mean 32–47) [12, 70, 71]. Although, IDH-wild-type and IDH-mutant gliomas are histologically similar, numerous groups have reported that gliomas harboring IDH mutations represent a distinct disease entity that arises from a different cell type and occur in the presence of other genomic abnormalities, such as TP53 mutation or 1p/19q chromosome deletion, and happen mutually exclusively in gliomas with EGFR amplification and chromosome 10 loss [17]. Moreover, IDH-mutant tumors have also been linked with substantial epigenetic changes, such as DNA methylation disorders, which harbor a striking pattern of hypermethylation of certain DNA promoter regions termed as glioma-CpG island methylator phenotype (G-CIMP) [72]. It has been shown that 78% of G-CIMP+ tumors carry IDH1 mutations [73], and 98% of these malignancies are positive for IDH2 [74].

The wild-type IDH gliomas including pilocytic astrocytomas and primary GBMs are independent of the IDH pathway (G-CIMP−). Conversely, most grades II and III gliomas and secondary GBMs share IDH mutations (G-CIMP+). Up to 87.5% of G-CIMP+ tumors represent a proneural gene expression subtype and are usually found in younger patients (mean age at diagnosis: 36 years versus 59 years) [73, 75]. Moreover, they also carry a better prognosis than the IDH-wild-type gliomas of the same histological grade [76]. In addition, Beiko et al. demonstrated that IDH1 mutation status is associated with the benefit of surgical resection in malignant astrocytic gliomas (WHO grades III and IV). More aggressive resection involving the whole tumor (both enhancing and nonenhancing part) correlated with better prognosis in patients with IDH1 mutant GBMs rather than with wild-type IDH1. In the case of the latter, no prognostic benefit was observed after the resection of nonenhancing part [77].

Importantly, since the presence of IDH mutations has been shown to correlate with better OS and progression-free survival (PFS) in GBM patients, this aberration became the only molecular marker included in the updated 2016 WHO classification of astroglial brain tumors [8, 78]. Furthermore, it prompted efforts to develop inhibitors of the mutated IDH protein for therapeutic purposes. So far, these new drugs have been reported to induce differentiation in preclinical models, and clinical proof of concept has been achieved in early phase I trials (NCT02074839 and NCT01915498) using AG-120 and AG-221 in adults with relapsed or refractory acute myelogenous leukemia [79–82]. These promising early results are now driving expansion of these trials into solid tumors. Phase I dose escalation studies of AG-120 in patients with IDH-mutated gliomas and other solid tumors (NCT02073994) and of AG-221 also in patients with IDH-mutated gliomas (NCT02273739) are now open. Further work is urgently needed to determine the most appropriate IDH mutation detection technique to facilitate early identification of patients who may benefit from these novel therapies. At the moment IDH mutations are evaluated by immunohistochemistry, standard sequencing, or genotyping methods. Furthermore, 2-HG level has been noninvasively assessed in patients with glioma using magnetic resonance spectroscopy and proved to be a useful biomarker in monitoring treatment response [82].

## 8. MGMT Methylation

Promotor of MGMT encoding *O*_6_-methylguanine-DNA methyltransferase is a DNA repair enzyme which can effectively protect cells against alkylating agents (e.g., TMZ and CCNU) through preventing G:C→A:T gene mutations [83]. Disorders of MGMT promoter methylation are associated with transcriptional silencing of the MGMT gene and loss of MGMT expression that results in decreased DNA repair and retention of alkyl groups, thereby allowing alkylating agents to be more effective in patients with MGMT promoter hypermethylation.

MGMT promoter methylation is more often found in secondary GBMs than in the lesions they derive from, such as low-grade gliomas and primary GBMs (75% versus 48% versus 36%) [11, 84]. Recently, a number of clinical trials have shown that MGMT methylation corresponds to greater PFS and OS in patients who are treated with alkylating agents [83, 85–87]. Therefore, MGMT promoter methylation status represents one of the most relevant prognostic factors in GBMs and has been considered as a potent predictor of response to alkylating agents.

Furthermore, recent prospective randomized trials (NOA-08), the Nordic trial, and RTOG 0525 have shown MGMT promoter methylation can also be a useful predictive biomarker to stratify elderly GBM patients for RT versus chemotherapy with alkylating agents. Several studies have demonstrated that patients with tumors with methylated MGMT promoter had a survival benefit when treated with TMZ and radiotherapy, compared with those who received RT only, whereas patients with MGMT promoter-unmethylated tumors had no survival benefit from chemotherapy, regardless of whether it was given at diagnosis together with RT or as a salvage treatment [88–92]. Consequently, it has been suggested that elderly GBM patients eligible for either RT or TMZ should undergo MGMT promoter methylation testing prior to the clinical decision being made. In addition, MGMT promoter methylation was associated with greater PFS and improved of OS in patients with recurrent GBMs (Director trial) [93]. These findings highlight the necessity for different therapeutic approaches in patients with GBMs depending on their MGMT status and introducing MGMT biomarker assessment into routine clinical practice.

## 9. Immune Checkpoints 

In addition to mutations in cell signaling and growth proteins, part of the aggressive nature of the GBM is related to its ability to escape immune system surveillance. GBM has established a mechanism of dampening the immune response by expressing immunosuppressive cytokines (e.g., prostaglandin E2 and transforming growth factor-*β*) and increasing activation of T-regulatory cells [94]. This suppressive immune microenvironment is manipulated by two important checkpoint proteins, cytotoxic T-lymphocyte antigen 4 (CTLA-4) and programmed cell death protein 1 (PD-1) [95]. CTLA-4 is exclusively upregulated on T cells and negatively regulates the early stages of T-lymphocyte activation by competing with the costimulatory molecule CD28 at binding the B7 ligands. In contrast, PD-1 is expressed by B, natural killer and dendritic cells as well as activated monocytes and tumor-infiltrative macrophages in addition to T cells [96, 97]. Moreover, PD-1 regulates immunity at multiple phases of the immune response effecting T-lymphocyte activity in the peripheral tissues [98]. TCGA analysis has reported high mRNA expression level of PD-L1, a PD-1 ligand, and CTLA-4 in mesenchymal GBMs suggesting a correlation between these immune checkpoint proteins and severity of GBM [99]. Yet, the prognostic value of these immune checkpoints in GBM is still controversial. Berghoff et al. performed a study on 117 GBM patient samples and found no correlation between PD-L1 and survival [100]. Liu et al. have revealed that PD-L1 can have both a positive and negative effect on GBM patient survival depending on the glioma subclass, on expression levels of PD-L1 regulatory molecules, and most importantly on the cell type that expresses PD-L1 in the tumor microenvironment [101]. But most current clinical studies have demonstrated that PD-1 and/or PD-L1 are immunohistochemically detectable in the majority of GBM samples and PD-L1 gene expression significantly correlates with molecular GBM subtypes (mainly mesenchymal) [100]. Additionally, Nduom et al. have shown that PD-L1 in GBM patients is overexpressed in a small subpopulation, where higher expression of PD-L1 is correlated with worse outcome [102].

With the dramatic success of checkpoint inhibitors (e.g., nivolumab and ipilimumab targeting PD-1 and CTLA-4, resp.) in melanoma, brain metastases, and lung and kidney cancers, hope has increased regarding the potential activity of these drugs in glioma [103–106]. Multiple clinical trials of these checkpoint inhibitors in GBMs and recurrent lower-grade gliomas are currently in progress (e.g., NCT02017717). These studies will determine whether this approach has any beneficial effects for these patients. So far, data from a phase III trial (NCT00045968) using a dendritic cell vaccine (DCVax-L) has indicated that monitoring CTLA-4 expression may predict survival in GBM patients, indicating there may be a role for CTLA-4 as a novel biomarker for treatment response [107, 108]. Furthermore, the RTOG is also planning a randomized phase II/III trial to test ipilimumab in combination with TMZ in patients with newly diagnosed glioblastoma [107]. Taken together, current findings indicate that the complexity of tumor microenvironment poses a major challenge to the development of immunotherapy approaches for GBMs and proper stratification of CTLA-4/PD-1/PD-L1-positive and negative patients will be important criterion for high-quality clinical trials in GBMs.

## 10. Imaging Biomarkers

The role of histopathology, proteomics, and next-generation sequencing methods as a standard reference for assessment of GBM progression is increasingly being challenged. In addition to invasiveness and sampling bias they do not address inter- and intratumor heterogeneity. Therefore, in contrast to conventional evaluation of ex vivo tissue specimens, development of imaging biomarkers for monitoring tumor response following therapeutic interventions could greatly improve individual patient management.

Currently there are no clinically approved imaging biomarkers for GBM. However, advanced functional imaging techniques including diffusion-weighted magnetic resonance imaging (DW-MRI) with apparent diffusion coefficient (ADC) mapping, dynamic susceptibility-weighted contrast-enhanced perfusion imaging, MR spectroscopy (MRS), and positron emission tomography (PET) have recently demonstrated a great potential for identifying distinct phenotypes of GBM tumors. While these results are promising, there is a large variation in sensitivity and specificity reported, which likely was a result of small sample size in some of these studies, and differences in acquisition protocols, as well as reference standards that have been used [109–111].

Nevertheless, several reports have highlighted that utilizing genomic and imaging data may improve the selection and implementation of the appropriate treatment for targeting the unique biology of GBM tumors and the detection of early treatment failure. For example, evaluation of 2-hydroxy-glutarate (2-HG) by proton MRS has been reported to correlate with the IDH1 or IDH2 mutations in the tumor [112] indicating that upregulated levels of 2-HG in IDH-mutated gliomas have the potential in the future to provide important diagnostic and prognostic information. Additionally, numerous MRI parameters such as a high ratio of contrast enhancing tissue to necrotic tissue (≥1), lower ADC values, increased T2 to contrast enhancing volume, deceased T2 border sharpness, and elevated relative cerebral blood volume (rCBV) have been reported as being predictive of EGFR amplification [113–115]. rCBV measurements have also shown to be a good predictive factor for the malignant degeneration-free survival, PFS, and OS as well as discrimination of tumor recurrence and nonneoplastic contrast enhancing tissue after radiotherapy in low-grade gliomas [116, 117].

Furthermore, an increase in tumor blood volume has been associated with EGFR amplification, PTEN deletion, and normal unmethylated MGMT [118–120].

Many of these MRI features are also essential in monitoring the clinical effectiveness of treatment regimens. Larsen et al. reported nearly 100% sensitivity and specificity using calculated CBV, which is comparable to those achieved by ^18^F-fluorodeoxyglucose (^18^F-FDG) on the same patients [121]. Besides, tumor ADC value has been shown to be a useful indicator for predicting response to bevacizumab. For example, in a large cohort of 97 bevacizumab-treated patients with recurrent GBM, low ADC was associated with a poor outcome in post hoc analysis from the multicenter randomized, phase II BRAIN study [122]. However, larger studies are needed for this imaging biomarker to become universally accepted. Also, functional diffusion mapping based on ADC values determined prior to and after radiochemotherapy were shown to correlate with both survival benefit and longer PFS in GBM patients [123].

Aside from MRI, a number of PET radiotracers have been evaluated as potential imaging biomarkers which may offer additional insight into brain tumor pathophysiology. Currently, ^18^F-FDG is the most frequently used PET radioligand, but it has limited capabilities for GBM imaging due to elevated glucose uptake in the brain compared to other tissues, which results in low-grade tumors, small tumors, and tumors with early recurrence remaining undetected. Therefore, for the past few years other PET ligands have been assessed including radiolabeled amino acids and their aromatic analogues (e.g., ^11^C-methionine (^11^C-MET), ^18^F-flouroethyltyrosine (^18^F-FET), and 3,4-dihydroxy-6-^18^F-fluoro-L-phenylalanine (^18^F-FDOPA)), and hypoxia agents (e.g., ^18^F-fluoromisonidazole (^18^F-FMISO)) since they overcome the limitations of ^18^F-FDG providing much higher tumor/background contrast. The amino acid PET tracers have attracted most of the attention due to the fact they enter the brain via amino acid transporters allowing visualization of both low- and high-grade gliomas regardless of integrity of the BBB [124, 125]. Kim et al., have demonstrated that among several clinical and metabolic factors, ^11^C-MET uptake is associated with poorer patient survival indicating a prognostic value of this tracer in glioma patients [126]. Pauleit et al. found that there is increased ^18^F-FET uptake in nonenhancing tumor areas which are difficult to delineate using MRI [127]. In addition, Fueger et al., have shown that imaging using ^18^F-FDOPA could differentiate between low- and high-grade gliomas and that tracer uptake correlated with tumor proliferation in newly diagnosed gliomas, but not in previously treated recurrent tumors [128]. A study of 22 participants with GBM has shown association between preradiation volume and degree of tumor hypoxia as measured by ^18^F-FIMSO and a shorter time to tumor progression and decreased survival. These promising results indicate that hypoxia imaging may also serve in the future as an early biomarker of radiation resistant tumor regions and provide insight into radiotherapy planning for patients with GBM [129]. While current data highlights the potential of molecular imaging biomarkers for the evaluation of treatment response and survival, further prospective studies are needed to evaluate their clinical impact. What is needed is an integration of comprehensive genomic information together with imaging data that will not only strengthen our understanding of heterogeneity in GBM's genetics, metabolomics, or epigenetics, but also provide an opportunity to identify robust predictive biomarkers that could improve therapeutic outcome and minimize drug resistance.

## 11. Conclusions

Although numerous challenges remain, recently substantial progress has been made in the molecular characterization of diffuse gliomas, providing useful insights into the development of more effective targeted therapeutics. Even though some of these therapies, including IDH or RTK pathway inhibitors, have so far produced limited or no therapeutic efficacy in phase III trials, our improved understanding of their mechanisms of action has helped to determine how to better incorporate their use in existing treatment paradigms. Importantly, based on the challenges these drugs have initially presented, innovative clinical trials have been designed evaluating different therapeutic strategies. The detailed description of these regimens was beyond the scope of this review but, as briefly mentioned in each paragraph, recent clinical reports are very promising. For example, given the potential to manipulate or enhance the immune system machinery to attack and kill tumor cells, immunotherapy has shed new light on and generated a lot of excitement in the treatment of GBM, especially with clinical trials that are currently underway. Phase I/II trials testing DCVax-L in patients with newly diagnosed GBM showed significant increase in the median life expectancy [6]. Furthermore, clinical trials based on either retroviral or adenoviral vectors have demonstrated that the herpes simplex virus-1 thymidine kinase (HSV1-TK)/ganciclovir (GCV) system HSV1-TK/GCV is well tolerated. However, due to immune suppression mechanisms present in GBM microenvironment, the study has not shown as expected significant therapeutic benefit [130].

Therefore, against this background, there is an urgent need to incorporate the status of known biomarkers into the routine clinical practice which may assist not only in patient selection, but also in the adjustment of treatment schedule based on the patient-specific biology. The biggest challenge lies in better understanding of GBM heterogeneity and the ability to successfully translate the vast amounts of data generated by large-scale, next-generation sequencing, and single tumor cell sequencing, as well as genomic and molecular imaging analyses into a clinically applicable format. Furthermore, appropriate combination of novel targeted and immunotherapeutic approaches that are biomarker driven will hopefully improve the management and lead to more durable responses in GBM patients.

## Figures and Tables

**Table 1:** Molecular biomarkers in GBM.

p53 signalling altered in 87%	MDM2 (amplification in 14%)
RTK/RAS/PI3 signalling altered in 88%	PI3K (mutation in 15%)
RB signaling altered in 78%	CDK4 (amplification in 18%)

**Table 2:** Major biomarkers relevant to the management of patients with glioblastoma.

Type of biomarker	EGFR mutation/ amplification	MGMT promotor methylation	IDH1/IDH2 mutation	Imaging	Reference
Diagnostic	EGFRvIII highly correlates with glioma subtypes. Real-time monitoring via typing of microvesicles with EGFR specific RNA.	Help to distinguish true progression and pseudoprogression in patients with newly diagnosed GBM treated with surgery followed by radiochemotherapy.	Differentiate between primary and secondary GBM. IDH-mutant diffuse gliomas and nonanaplastic reactive gliosis distinction.	Detection of specific molecular abnormalities. For example, EGFRvIII, MGMT promotor methylation, and 2-HG which correlates with IDH mutation.	[9, 76, 112–115, 118–120, 131–134]

Development status	+/under evaluation	Under evaluation	+	+	

Prognostic	Better prognosis with (i) EGFRvIII + Ki64 20% or less, (ii) EGFRvIII + normal PTEN, (iii) EGFRvIII + methylated MGMT promotor.	Better OS and PFS (probably with IDH mutations) in malignant gliomas treated with radio- and/or chemotherapy.	Better OS and PFS	MRI: extent of tumor edema and necrosis has negative correlation with OS. PET: ^11^C-MET uptake is associated with poorer patient survival.	[32–36, 43, 44, 76, 78, 83, 85, 93, 126, 135–140]

Development status	+/under evaluation	+	+	Under evaluation	

Predictive	Possible biomarker for vaccine-based treatment.	Predicts response to chemotherapy with alkylation agents and radiotherapy. Correlate with better response to TMZ in (i) newly diagnosed GBM with TMZ as a first-line treatment, (ii) recurrent GBM, (iii) elderly patients.	IDH1 mutation is independently associated with complete resection in patients with malignant gliomas treated with surgery. Complete surgical resection is associated with improved survival in patients with IDH1 mutation. Absence of mutation suggests predictive role of MGMT promotor methylation for PFS in patients treated with chemotherapy.	Functional Diffusion Maps (fDMs) predicts PFS and OS in patients treated with radiochemotherapy. ADC predicts better response to bevacizumab combined with chemotherapy. Association between hypoxia level (measured by ^18^F-FIMSO and radiotherapy response.	[77, 86–91, 93, 122, 123, 136, 140, 141]

Development status	Under evaluation	+	Under evaluation	Under evaluation	

OS, overall survival; PFS, progression-free survival.
